# Preliminary Study on the Effect of an Early Physical Therapy Intervention after Sentinel Lymph Node Biopsy: A Multicenter Non-Randomized Controlled Trial

**DOI:** 10.3390/ijerph18031275

**Published:** 2021-01-31

**Authors:** María Jesús Muñoz-Fernández, Esther M. Medrano-Sánchez, Beatriz Ostos-Díaz, Rocío Martín-Valero, Carmen Suárez-Serrano, María Jesús Casuso-Holgado

**Affiliations:** 1 Department of Physiotherapy, Faculty of Nursing, Physiotherapy and Podiatry, University of Seville, C/Avicena s/n, 41009 Seville, Spain; mariamufe_93@hotmail.com (M.J.M.-F.); beatrizostosdiaz@hotmail.es (B.O.-D.); csuarez@us.es (C.S.-S.); 2 Department of Physiotherapy, Faculty of Health Sciences, University of Malaga, Arquitecto Francisco Peñalosa 3, Ampliación de Campus de Teatinos, 29071 Malaga, Spain; rovalemas@uma.es

**Keywords:** breast neoplasms, sentinel lymph node biopsy, physical therapy, adverse effects, tissue adhesions

## Abstract

Selective sentinel lymph node biopsy (SLNB) represents a minimally invasive surgery in patients with breast cancer. The purpose of this study was to explore the possible effect of an early physiotherapy intervention for the recovery of the upper limb and the surgical scars after SLNB in comparison with usual care. A total of 40 patients were enrolled in either the control group (*n* = 20) or the experimental group (*n* = 20). The intervention group performed an early physiotherapy program based on functional exercises, scar manual therapy, and educational tips. The control group received usual care. Shoulder range of motion (ROM), grip strength, upper limb pain and disability (SPADI), scar recovery (POSAS), myofascial adhesions (MAP-BC), quality of life (EORTCQLA-BR-23) and the presence of axillary web syndrome (AWS) and lymphoedema were assessed at baseline and immediately after intervention. A follow-up period of 6 months was performed for lymphoedema surveillance. Between groups significant differences in favor of the intervention were found for ROM (r = 0.43), grip strength (r = 0.32), SPADI (d = 0.45), POSAS (d = 1.28), MAP-BC (d = 1.82) and EORTCQLQ-BR 23 general function subscale (d = 0.37) (*p* < 0.05 for all variables). Our results suggest that an early physical therapy program seems to be more effective than usual care in women after SLNB. However, results should be interpreted with caution and future randomized trial with a larger sample size is needed.

## 1. Introduction

Selective sentinel lymph node biopsy (SLNB) is considered a safe technique for axillary lymph node evaluation without the need for other more invasive techniques as, for example, axillary lymph node dissection [1,2]. At present, this type of surgery, in conjunction with breast-conserving surgery as well as neoadjuvant therapy, is the treatment of choice of those women diagnosed with breast cancer in earlier stages with low metastasis suspicions [3,4].

However, although it is a less invasive surgical intervention, negative physical consequences may follow. The scientific literature proves that after SLNB, women are at risk of development of arm lymphedema, disability of shoulder function, pain, and decreased muscle strength, and quality of life [5,6,7]. In a 1-year follow-up study, 100 negative-node SLNB patients were assessed, and half of them had pain and impaired shoulder function [8]. Glowacka et al. [9] compared the long-term sequelae after breast-conserving surgery depending on the type of axillary surgery (sentinel lymph node biopsy or axillary lymph node dissection), and their results revealed that both groups had limited range of motion of the shoulder joint, sensation disturbances and winged scapula sign.

Another common condition observed after breast cancer surgery is the axillary web syndrome (AWS), which is a tense, non-erythematous painful band. It is palpable and visible under the skin and may appear from the armpit to the medial upper limb with extension to the antecubital fossa [10,11]. Although SLNB is linked to a lower risk of suffering AWS compared to the axillary lymph node dissection (ALND) technique, it is not free of this complication, and early post-surgical care is advisable in this population [10].

Similarly, lymphedema is still a problem in SLNB patients. A systematic review demonstrated that lymphedema is a nonnegligible complication in patients with SLNB-negative breast cancer, since the incidence of lymphedema in this population ranged from 0 to 63.4% [12]. In a 4-year follow-up, Yen et al. [13] reported that lymphedema was developed in 7% of the patients over 65 years who underwent SLNB.

Surgical scars have also a negative impact on the quality of life of patients operated on breast cancer. Recently, Gass et al. [14] reported, through a national survey in the United States, that the majority of women feel negatively affected by their breast cancer surgery scars. In addition, 67% of these women did not like the scar location. Breast cancer surgery may also cause scar adhesions and fibrous tissue [15]. Myofascial techniques improved pain, mobility, and functionality related to breast surgery scar adhesions when applied from 4 months to 3 years after surgery [16,17], but the scientific literature about an early treatment of physiotherapy applied to the scar tissue after breast cancer surgery is limited.

Several studies have indicated a negative physical impact on patients after SLNB and ALNB, and therefore, treatment to alleviate these symptoms in both types of surgery is suggested. Nevertheless, research investigating the impact of an early rehabilitation program after SLND is scarce [18]. At present, there is a previous study that shows the feasibility of the proposed intervention in a single group [19].

Due to the absence of previous research establishing whether, compared to usual care, an early physiotherapy intervention improves the recovery of upper limb and surgical scars after SLNB, the main purpose of this pilot study was to collect preliminary data on the effects of the aforementioned intervention [19] in order to plan a larger randomized controlled trial.

## 2. Materials and Methods 

### 2.1. Study Design

This is a quasi-experimental non-randomized controlled pilot trial. Randomization was not possible because the “Virgen del Rocio” hospital (Sevilla, Spain) offers early treatment of physiotherapy to all women who undergo SLNB, but the efficacy of this intervention has not been proven. It was therefore decided to select the control group of the nearby “De la Merced” hospital (Sevilla, Spain). 

The implemented study design was executed according to Consolidated Standards of Reporting Trials (CONSORT) statements [20]. All research procedures were approved by the Local Ethics Committee on Human Research (PEIBA nº 1176-N-17). The study was prospectively registered in the Australian New Zealand Clinical Trials Registry (Trial Id: ACTRN12618000719235).

Informed consent was signed by all participants prior to surgical intervention, following the recommendations of the Declaration of Helsinki and the legal regulations in force in Spain regarding clinical research, especially Law 14/2007, of July 3, on biomedical research. The patients were also informed in a clear, precise, and sufficient way of all the aspects included in the study.

### 2.2. Participants and Setting

During a period of eighteen months, participants were recruited in the two breast surgery units from the hospitals mentioned above. Inclusion criteria were as follows: (1) between 18 and 90 years old, (2) diagnosed with breast cancer, (3) intervened using the sentinel lymph node biopsy technique, (4) medical clearance of participation, (5) signed informed consent. The exclusion criteria were defined as follows: (1) history of ipsilateral cancer recurrence, (2) chronic disease or orthopaedic issues that would interfere with the ability to participate in this rehabilitation program, (3) existence of psychiatric disorders.

The day before surgery, the researchers contacted in person with patients scheduled for SLNB in both hospitals, and all subjects who met the inclusion criteria were then evaluated (T0 or baseline). Subjects were then enrolled in the control group (Hospital 1) or the experimental group (Hospital 2). After surgery, patients were evaluated three times within 1 month: after hospital discharge (T1), after the stitches were taken out (T1 scar-related variables) and at the end of the intervention, or at 1 month after surgery in the case of the control group (T2). There was also a follow-up period of 6 months (T3). All evaluations were carried out by two experienced physical therapists (Table 1). Equal materials were used, and before starting the patient recruitment, several training sessions were performed.

### 2.3. Outcome Measures

The primary outcome measures were upper limb function (range of motion, strength, pain and disability) and scar recovery (state of scar and myofascial adhesions). Secondary outcomes were quality of life and the incidence of axillary web syndrome and lymphedema.

#### 2.3.1. Range of Motion (ROM)

To assess mobility, we used goniometry. The goniometer is the standard measure to assess the range of motion. Subjects were asked to move their arms in flexion, extension, abduction, and internal and external shoulder rotation. Maximum ROM was considered to be 180° for movements of flexion and abduction, 45° for extension, 100° for internal rotation, and 80° for external rotation. A single index was calculated as the percentage of the global movement [21].

#### 2.3.2. Strength

Grip strength was measured in kilograms using a calibrated hand dynamometer (JAMAR). The assessment was repeated three times with the hand of the affected side, and the average of the three tests was used for the main analysis [22].

#### 2.3.3. Pain and Disability

To assess the pain and disability of the upper limb, we used the Shoulder Pain and Disability Index (SPADI) scale [23]. The SPADI contains 13 items that assess two domains, a five-item subscale that measures pain and an eight-item subscale that measures disability. This scale is validated in Spanish and also specifically in the breast cancer population [24,25]. Each scale’s item is scored by a numeric rating scale that ranges from 0 (no pain/no difficulty) to 10 (worst pain imaginable/that difficult it required help). A higher score indicates greater pain-related disability. 

#### 2.3.4. State of Scar

The Patient and Observer Assessment Scale (POSAS) was used to assess the recovery of breast and axillary scars [26,27]. It is composed of two separated six-item scales (the observer and the patient scales). The observer scores six items: vascularization, pigmentation, thickness, surface roughness, pliability, and surface area. The patient also scores six items: pain, pruritus, color, thickness, relief and pliability. All items are scored on the same polytomous10-point scale, where a 1 is awarded when the scar is the closest to “normal skin” and up to 10 when the scar is in the worst possible condition. All items are added to obtain a final score (0–60); the higher the score, the worse the condition of the scar. 

#### 2.3.5. Tissue Adhesions/Myofascial Adhesions

The evaluation tool for Myofascial Adhesions in Patients after Breast Cancer (MAP-BC evaluation tool) was used to assess the degree of adhesions that exist in the scar tissue and its surroundings. This scale was developed to quantitatively evaluate myofascial adhesions in patients with breast cancer [28,29]. The degree of adhesions is scored at three levels of depth (skin, superficial and deep) and in turn on a scale of four points in each area (between 0: no adhesion and 3: very strong adhesions). The areas to be valued are as follows: axillary scar, breast scar/mastectomy scar, pectoralis region, frontal chest wall, lateral chest wall, axilla and inframammary fold. The final score is obtained with the sum of the three levels of each area; the minimum score is 0 and the maximum score 63.

#### 2.3.6. Quality of Life

To measure the quality of life, a specific quality-of-life questionnaire was used, namely the Spanish version of the EORTCQLQ- BR-23 cancer-specific quality-of-life [30]. It is composed of 23 items divided into four functional scales (body image, sexual functioning, sexual enjoyment, future perspective) and four symptoms scales (systematic therapy side effects, breast symptoms, arm symptoms, and upset by hair loss). Scores vary from 0 (worst) to 100 (best) for function and from 0 (best) to 100 (worst) for symptoms.

#### 2.3.7. Axillary Web Syndrome

The presence of axillary web syndrome (Yes/No) was evaluated by observation and palpation by the evaluators. Physical examination was performed as suggested in previous research: patient in a supine position with the elbow extended and the shoulder maximally abducted. The evaluator both visualizes and palpates for cords including the axilla, down the upper arm from the axilla to and across the antecubital space and down the forearm to the base of the thumb [10,11].

#### 2.3.8. Lymphoedema

The presence of lymphedema (Yes/No) was evaluated via a telephone survey 6 months after the surgery. Women were classified as having self-reported lymphedema if they answered “yes” to the following question: ‘‘Since your breast cancer surgery, has a doctor ever told you that you have lymphoedema or arm edema?’’ [13].

### 2.4. Interventions

#### 2.4.1. Experimental Group

**Figure 1:**
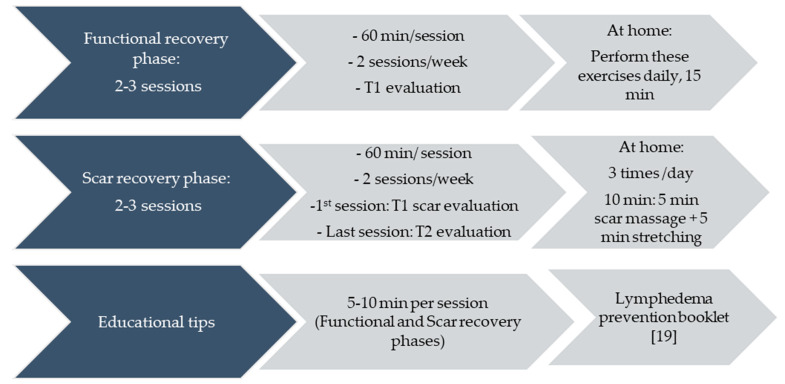
Temporal distribution of the intervention.

The intervention group received a supervised early physical therapy intervention based on functional recovery exercises and scar treatment with manual therapy in conjunction with educational tips on the management of the upper limb and the scar at home. The intervention consisted of four to six sessions within a month, depending on the evolution of the patient (Figure 1). This temporal distribution was agreed with the surgeons, aiming to conclude our intervention prior to the first post-surgery revision. The first session occurred up to a mean of 8.3 days after the breast cancer surgery.

##### Functional Recovery

The objective of this stage was to normalize muscular tone, improve lymphatic drainage and restore the complete mobility of the upper limb, minimizing any residual limitation. 

##### Functional Recovery Exercises

It was performed as a program of exercises focusing on functional recovery and centered on lymphoedema prevention, as well as on postural hygiene and individualized exercises depending on the patient’s progress. These exercises consisted of respiratory movements, particularly diaphragmatic breathing, accompanied by upper limb movements, stretching, and progressive assisted active exercises. With these exercises, global functionality was treated, as well as muscular work, minimizing paresthesia symptoms in the upper limbs. A detailed description of these exercises is available online (Table S1).

At home, all exercises should be done three times a day and should not last more than 10 min in total. 

##### Scar Treatment

The first session took place at least 2 days after stitches removal. The women were taught how to clean the scar so that the poles could be gently removed (Vaseline, shower, drying antiseptic application). The patient was told that she should verify that the upper right underwear should be tight but not too tight because, in addition to discomfort, it could cause the accumulation of fluid in the sub axillary area. 

##### Scar Exercises

**Figure 2:**
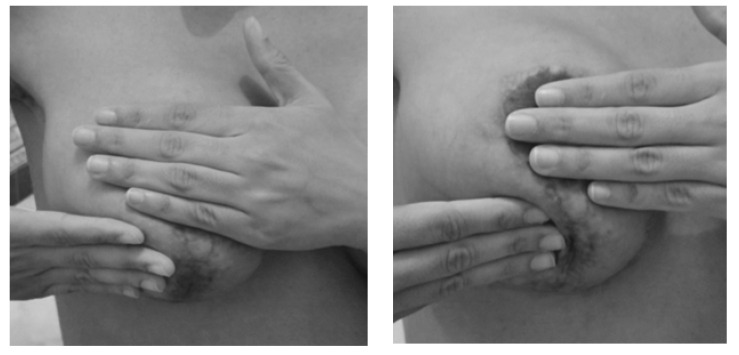
Manual therapy on breast scars.

**Figure 3:**
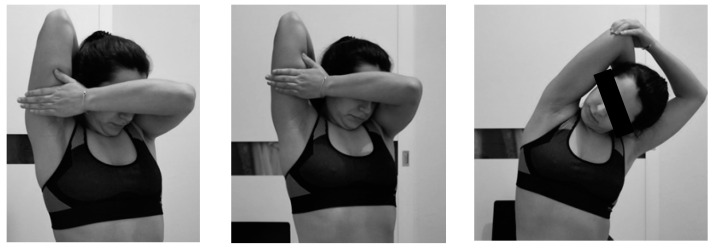
Stretching exercises in the scar recovery phase.

The surrounding areas of the scar were normalized by manual therapy and stretching in order to provide elasticity and prevent adhesions. The hardened areas were emphasized (Figure 2). At the end of the sessions, the armpit was stretched, remembering that it could cause discomfort, but not pain (Figure 3). A detailed description of the scar treatment is available online (Table S1).

At home, patients had to repeat scar treatment three times a day for a period of maximum 10 min. 

##### Educational Tips for Lymphoedema Prevention

The educational intervention has been described in detail elsewhere [19]. Educational tips centered on lymphedema prevention and postural hygiene were given to the participants. They were provided with information on how to improve the lymphatic system, as well as how to avoid risks that can contribute to its depletion. Both verbal and graphic information was used.

#### 2.4.2. Control Group

The control group received usual care based on basic medical recommendations in written form before hospital discharge. Evaluation was implemented in the nurse and the surgeon office. 

### 2.5. Statistical Analysis

Based on the recommendations for the design and analysis of pilot studies [31], we aimed to recruit 40 subjects. Recruitment closed when 20 participants, which had been included in each of the two study arms, completed post-intervention follow-up.

Data analysis was carried out using the Statistical Package for the Social Sciences (SPSS, version 20.0, IBM Corp., Armonk, NY, USA). The Shapiro-Wilk test was used to determine whether there was a normal distribution. Thus, categorical variables were assessed using the chi-square test. The continuous variables were compared using the t-test (for parametric variables) or the Wilcoxon and U Mann-Whitney test (for non-parametric variables) for non-paired samples analysis. Effect size was calculated using Cohen’s d statistic for parametric outcomes and by the statistic r (Rosenthal) for non-parametric. We adopted a *p* < 0.05 as the statistical significance limit. 

## 3. Results

**Figure 4:**
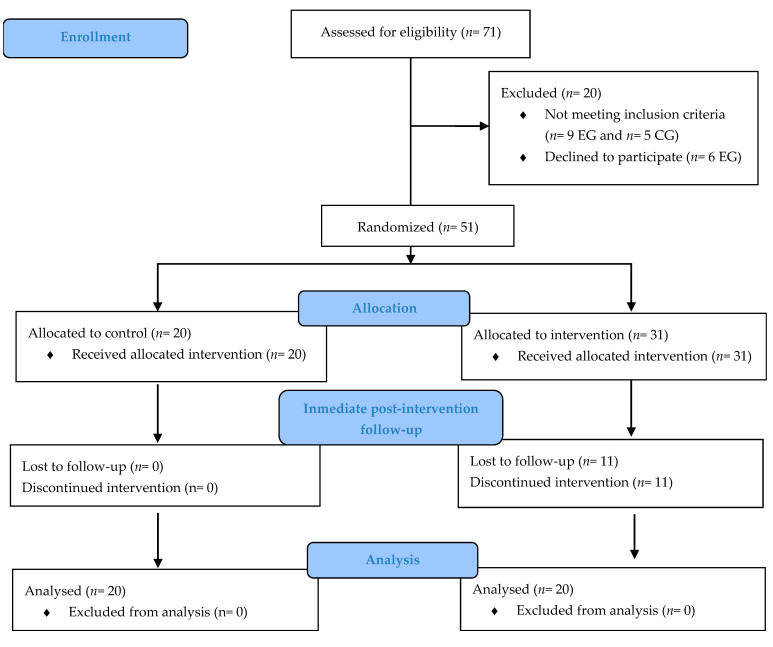
Flow chart of the study.

During the study period, 71 women were recruited in both hospitals. Of these, 25 women were recruited from the control group and 46 from the experimental group; 14 women were excluded for not meeting the inclusion criteria and 6 declined to participate. Finally, 20 women were allocated to the control group and no one was lost during the follow-up nor excluded from analysis. In total, 31 participants were allocated to intervention, and 11 women were lost during the follow-up period. Finally, 20 women were analyzed in the experimental group. Drop-out patients were not included in the analysis since no data were available after intervention. When they were contacted, the main reason for drop-out was the time they needed to travel to the hospital. Figure 4 shows a more detailed trial profile.

### Preliminary Data of Clinical Outcomes

The participants’ basic demographics and clinical-surgical characteristics are shown in Table 2. At baseline, both groups were homogeneous, with no differences observed between the two groups except for the variable grip strength.

Intra-group differences before and after the intervention are shown in Table 3. In the case of the experimental group, significant improvements were found for the variables global shoulder ROM (*p* = 0.003), global SPADI (*p* = 0.001), state of scar (*p* = 0.000), myofascial adhesions (*p* = 0.000), and quality of life-subscale general function (*p* = 0.013). Contrary, no significant differences were found for grip strength (*p* = 0.113) or quality of life-subscale general symptoms (*p* = 0.072). In the experimental group, two women developed axillary web syndrome (10%), and none developed lymphedema 6 months after surgery. On the other hand, in the control group, significant differences were observed for the variables myofascial adhesions (*p* = 0.002) and general function (*p* = 0.000). However, it must be noted that the statistical significance in the case of myofascial adhesions was due to a worsening of these adhesions at T2. For the remaining variables of the control group, there were no statistically significant changes. No woman developed axillary web syndrome (0%) or lymphoedema 6 months after surgery.

Between-group comparisons showed significant differences in favor of the experimental group for the variables global shoulder ROM (*p* = 0.006, r = 0.43)), grip strength (*p* = 0.041, r = 0.32), global SPADI (*p* = 0.046, d = 0.45), state of scar (*p* = 0.000, d = 1.28), myofascial adhesions (*p* = 0.004, d = 1.82) and quality of life-subscale general function (*p* = 0.011, d = 0.37); no significant differences were found for quality of life-subscale general symptoms (*p* = 0.296), axillary web syndrome (*p* = 0.244) and lymphoedema development (Table 3). No adverse or harmful events were reported in both groups.

## 4. Discussion

The aim of this pilot trial was to collect preliminary data on the effect of an early physiotherapy intervention for the recovery of the upper limb and the surgical scars in women after SLNB surgery in comparison with usual care. A total of 40 women from two hospitals were analyzed.

In general, our intervention was based on improving the short-term symptoms after SLNB, thereby preventing the long-term sequelaes that have been associated with this type of surgery [6,7,8,9,32]. The intervention proposed was in agreement with previous research focused on flexibility and mobility exercises and/or educational programs after ALNB. [33,34,35,36] The present study also agrees with the intervention of Koehler et al. [11] for AWS treatment, but in our case, it was used for preventive purposes.

Based on our results, a brief physical therapy intervention applied during the first month after surgery seems to be more effective than usual care for the recovery of the upper limb and scars in the population studied. Between-group differences in favor of the experimental group were observed for the global shoulder ROM, grip strength, global shoulder disability, recovery of scar, myofascial adhesions and quality of life (general function subscale). There were no statistical differences between groups for quality of life (general symptoms subscale) or the development of axillary web syndrome and lymphoedema.

Our results are in accordance with Scaffidi et al. [18], who concluded that early rehabilitation is necessary in women who have received SLNB and ALND since shoulder mobility can be affected, in addition to the possibility of developing lymphoedema. Contrary to our study, differences between experimental and control group were not found for ROM at 30 days of follow-up, and incidence of lymphedema at 6 months was significantly reduced in the experimental group. However, this study included both women who have received SLNB and ALNB, and the sample of women who had undergone surgery using the SLNB technique was relatively small.

Sato et al. [37] compared the effectiveness of a perioperative educational program for the recovery of the upper limb in patients with ALNB and SLNB in comparison with a control group for each surgery type. The intervention was based on providing guidelines to improve the mobility and strength of the affected arm after discharge. In the SLNB group, there was no observed significant improvement, and therefore, in contrast with our results, it was concluded that the intervention carried out was not effective for SLNB recovery. However, as mentioned before, the intervention was based on an educational program and therefore largely depended on the adherence to the treatment of each patient. Our intervention was based on sessions guided by physical therapists, in conjunction with educational advice. In agreement with our results, they did not report a significant difference in the appearance of lymphoedema, although it was assessed at three months, in contrast to 6 months in our study.

Regarding quality of life, our results could be explained by previous findings. For example, Peintinger et al. [32], in a longitudinal study, observed that SLNB did not have a high impact on the quality of life of women in the short term. Contrary, quality of life deterioration has been observed in the long term [7]. We hypothesized that maybe more time is needed for the perception of the possible impact of the surgery on the quality of life. Upper limb morbidity and lymphoedema incidences reported by the literature after SLNB are largely different from our results. De Groef et al. [8] showed that 1 year after surgery, 50% of sentinel node-negative breast cancer patients had pain, about 30% had decreased ROM, 8% had decreased handgrip strength and 49% presented with disability. These results indicate that breast surgery using the SLNB technique may also cause long-term adverse effects; nevertheless, in our study, these variables were restored to baseline at least for a short-term period (1 month after surgery). Similarly, Gebruers et al. [12] reported that the incidence of lymphedema at 6 months varied from 2% to 10%, while in our study, the incidence was 0% in both groups. In contrast, our incidence rates of AWS are in agreement with previous research reporting this complication in 18% of women after SLNB at 12 weeks [38]. However, although around 90% of the cords appear within 30 days of the surgery [10], such comparisons should be regarded with caution.

On the other hand, there are, to date, no studies assessing the effect of an early physical therapy intervention on the recovery of surgical scars. Our results suggest that early scar mobilization could be an advisable strategy for scar recovery after SLNB. It has been shown that in the long term, scars after breast surgery affect women in their quality of life [14], and therefore, early scar treatment could be taken into consideration.

Our study has, however, some limitations. First, it is a pilot study with a small sample size, although the role of pilot studies in health research, when used to plan a larger randomized controlled trial, has been pointed out [31]. Second, randomization was not possible, and patients were allocated by hospital setting. Despite this, patients were recruited from two hospitals from the same health management area. Third, evaluation was performed by two different non-blinded physical therapists, which could have influenced the outcomes. However, prior to the study, we organized consensus meetings, and equal materials were used. Therefore, a methodological risk of bias exists, and results should be interpreted with caution. Fourth, the drop-out rate in the experimental group was higher than that in the control group, which was mainly due to the distance to the hospital setting and was not related to the intervention. Fifth, a baseline difference was observed for handgrip strength, which could partly explain the part post-intervention results, although the trend of these differences makes it unlikely. Finally, a longer lymphoedema follow-up using an objective measurement, not only self-reported, would be of interest. The strengths and implications of our study are as follows: to our knowledge, this is the first controlled trial aiming to study the effect of an early physiotherapy treatment based on general function recovery, manual therapy scar treatment, and home-based recommendations in women after SLNB in comparison with usual care. Taking into account our results, this intervention could be applied in all cases after SLNB surgery. Furthermore, the intervention of the present study had no adverse effects, and due to the brief number of sessions, its economic cost is expected to be low. 

## 5. Conclusions

In conclusion, our results suggest that an early physical therapy program seems to be more effective than usual care for the recovery of the upper limb and the surgical scars after SLNB. In contrast, our results do not seem to support the hypothesis that early physiotherapy intervention prevents the development of lymphoedema or axillary web syndrome. However, these results should be interpreted with caution, and future research considering a larger sample size in a single-blinded randomized controlled trial is needed. A cost-effectiveness study would also be desirable.

## Figures and Tables

**Table 1:** Data collection process.

T0 Evaluation	T1 Evaluation	T2 Evaluation	6 Months Follow-Up
Upper limb function: Shoulder ROM Grip strength Upper limb pain and disability (SPADI) Quality of life (EORTCQLQ- BR-23)	Upper limb function: Shoulder ROM Grip strength Upper limb pain and disability (SPADI) Scar-recovery outcomes:State of scar (POSAS) Tissue adhesions (MAP-BC) Quality of life (EORTCQLQ- BR-23)	T1 measurements plus: Axillary web syndrome exploration Lymphoedema exploration	Lymphoedema surveillance

ROM: range of motion; SPADI: Shoulder Pain and Disability Index; POSAS: Patient and Observer Scar Assessment Scale; MAP-BC: Myofascial Adhesions in Patients after Breast Cancer tool.

**Table 2:** Basic demographics and clinical-surgical characteristics at baseline.

	EG (N = 20) Mean (SD) Median (Range)	CG (N = 20) Mean (SD) Median (Range)	*p* Values
Age (years)	59.25 (7.52)	64.15 (11.78)	0.125
BodyMassIndex	25.73 (4.31)	26.76 (3.17)	0.401
Ethinicity (C/L)	19/1	20/0	0.311
Type of BreastCancer DCIS IDC LCIS Others	4 15 1 0	1 14 1 4	0.098
Stage of BreastCancer IA IB IIA IIB IIIA Missing	11 1 3 1 1 3	15 1 2 2 - -	0.751
Type of breastsurgery (SUM/BCS)	2/18	5/15	0.215
Numbers of lymphnodesremoved ^a^	2 (0.75)	2 (1.75)	0.760
Positive LymphNodes ^a^	0.00 (0.00)	0.00 (0.00)	0.553
Side Involved Right Left	9 11	9 11	-
Involvedsidetohanddominance (Yes/No)	11/9	8/12	0.342
AdjuvantTherapy: chemotherapy (Yes/No)	4/16	5/15	0.705
Numbers of sessions of adjuvanttherapy ^a^	7.50 (5.50)	15.00 (8.00)	0.079
ROM ^a^	100.00 (8.55)	100.00 (0.00)	0.309
Gripstrength	10.35 (4.45)	18.85 (6.01)	0.000
Global SPADI ^a^	5.50 (17.50)	6.00 (15.25)	0.806
QoLFunction	54.17 (12.83)	54.90 (19.37)	0.889
QoLSymptoms ^a^	7.80 (13.89)	11.18 (25.80)	0.588

EG: experimental group; CG: control group; C: caucasian; L: latinamerican; DCIS: ductual carcinoma in situ; IDC: invasiveductual carcinoma; LCIS: lobular carcinoma in situ; ROM: range of motion; SUM: simple unilateral mastectomy; BCS: breast-conservingsurgery; QoL: quality of life. ROM: range of motion, SPADI: Shoulder Pain and Disability Index. ^a^: non-normal distribution (median, range and non-parametriccomparisons are reported).

**Table 3:** Comparison of the study outcome measures.

Outcomes	Experimental Group (*n* = 20)			Control Group (*n* = 20)			EG/CG *p*-Values	Effect Sizes
	Pre-Intervention T1	Post-Intervention T2	*p*-Values	Pre-Intervention T1	Post-Intervention T2	*p*-Values		
ROM ^a^	94.87 (26.92)	100.00 (0.00)	0.003	90.60 (29.49)	100.00 (29.91)	0.498	0.006	0.43
GripStrength ^a^	14.85 (9.18)	15.15 (6.67)	0.113	18.88 (6.45)	18.62 (6.54)	0.380	0.041	0.32
Global SPADI	32.30 (26.84)	14.55(14.95)	0.001	27.90 (28.84)	24.15 (25.88)	0.466	0.046	0.45
POSAS	25.90 (9.57)	9.00 (5.60)	0.000	21.30 (11.32)	20.70 (11.67)	0.709	0.000	1.28
MAP-BC	28.15 (9.66)	5.70 (5.33)	0.000	19.10 (11.04)	24.70 (13.79)	0.002	0.004	1.82
QoLFunction	47.71 (11.29)	53.72 (12.55)	0.013	44.59 (15.69)	58.78 (14.54)	0.000	0.011	0.37
QoLSymptoms	20.31 (14.40)	17.09 (10.89)	0.072	17.54 (10.42)	17.54 (12.32)	0.999	0.296	0.038
Axillary web syndrome	-	2 (10%)	-	-	0 (0%)	-	0.244	
Lymphedema	-	0 (0%)	-	-	0 (0%)	-	-	

ROM: range of motion; SPADI: Shoulder Pain and Disability Index; POSAS: Patient and Observer Assessment Scale; MAP-BC: Myofascial Adhesions in Patients after Breast Cancer. ^a^: non-parametric measures.

## Data Availability

The data presented in this study are available on request from the corresponding author.

## Supplementary Materials

The following are available online at https://www.mdpi.com/1660-4601/18/3/1275/s1, Table S1: Description of the exercises conducted in the functional recovery and scar treatment phases.
