# Supplementary material for: Preliminary Study on the Effect of an Early Physical Therapy Intervention after Sentinel Lymph Node Biopsy: A Multicenter Non-Randomized Controlled Trial
Source: Int J Environ Res Public Health. 2021 Jan 31;18(3):1275. doi: 10.3390/ijerph18031275 (PMC7908454; doi:10.3390/ijerph18031275)
Supplement: Supplementary file 1 [file ijerph-18-01275-s001.pdf]

Table S1. Description of the exercises conducted in the functional recovery and scar treatment phases.

Functional recovery exercises:

1. Patient sitting, with arms extended on the table, gradually lowering the body leaving the arms extended on the table. The patient should feel the axillary area stretching, thus leaving free and moving all the tissues around the area operated with scar tissue. The patient had to maintain the stretch for approximately 25-30 seconds and perform five repetitions.

2. Respiratory exercise. Patient sitting, the exercise was performed in three postures (posture 1: hands at the level of the navel, posture 2: hands at the level of the chest and posture 3: hands at the supramammary level); the women took a breath and put their hands to posture 1, when they expelled the air, they extended their arms completely, returned to take air and took the hands to posture 2, when they expelled it, they returned to extend the arms completely and finally took air and took the hands to posture 3, and when they expelled the air,, they raised their arms outstretched above their heads. This exercise should not cause pain; if there was pain, the women should raise their arms less, staying at the level where they only felt tension, and simply increased the tension in order to stretch all the tissues well; this way, the scar tissue was properly arranged and intervened. This exercise had to be repeated five times per posture; duration was according to the breathing of each patient.

3. Patients lying supine, interlocking their hands, and with their elbows stretched, they carried their arms until they touched the stretcher and felt tension. Just like in the previous exercise, if they felt pain before touching the stretcher, they should only go up until they felt tension. There were five repetitions, and the patients had to rise slowly and slowly return to the starting position.

Scar exercises:

1. Patients were taught manual therapy to treat their own scar or scars. With flat fingers, they had to massage the entire breast by pushing inwards and upwards, thus giving movement to the entire breast tissue. This also helped to drain the liquid after the operation. This exercise should not be done for longer than 2 minutes.

2. The next technique also consisted of manual therapy: with flat fingers, the edges of the scars had to be massaged in the direction of the scar and inward. The entire scar was bordered, and the hardest areas were touched. The technique should last for about 2 minutes.

3. Stretching 1. Raising the affected arm with the elbow flexed touching the neck, with the hand of the opposite arm at the level of the armpit, making an upward and inward thrust. The patient should notice tension and hold the position for about 30 seconds, with three repetitions.

4. Stretching 2. Raising the affected arm with the elbow flexed touching the neck; with the hand of the opposite arm at the level of the supra-axillary area, making an upward and inward thrust. The patient should notice tension and hold the position for about

30 seconds, with three repetitions.

5. Stretching 3. Raising the affected arm with the elbow flexed touching the neck; with the hand of the opposite arm at the level of the ipsilateral elbow, leaning towards the opposite side to the affection. The patient should notice tension and hold the position for about 30 seconds, with three repetitions.
